# Clinical evaluation of paraspinal mini-tubular technique vs. laminoplasty for spinal intradural extramedullary tumors: Study protocol for a multicenter, randomized controlled trial

**DOI:** 10.3389/fsurg.2022.1053885

**Published:** 2023-01-05

**Authors:** Rui Wang, Ze-Yan Liang, Yan Chen, Chun-Mei Chen

**Affiliations:** Department of Neurosurgery, Fujian Medical University Union Hospital, Fuzhou, China

**Keywords:** paraspinal minitubular technique, laminoplasty, randomized controlled trial, intradural extramedullary, multicenter

## Abstract

The development of minimally invasive surgery has promoted the use of the paraspinal mini-tubular technique (PMTT) for spinal tumors. The similarity of the efficacy of PMTT to that of traditional surgery remains unclear; randomized controlled trials (RCTs) have not been conducted to investigate this technique for spinal tumors. The conventional surgery used for such tumors is laminoplasty (LP). To compare the differences between the two surgical techniques, an RCT is significantly required. Therefore, a prospective, multi-center, non-inferiority RCT was designed to compare the safety and effectiveness of LP and PMTT for treating spinal intradural extramedullary (IDEM) tumors. Moreover, the availability of PMTT, including its advantages and disadvantages, surgical indications, procedures, complications, and prognosis, would be explored. Overall, 280 patients will be randomly allocated to the PMTT and LP groups in a 1:1 ratio. The trial hypothesis is that PMTT has superior or equivalent efficacy and cost-effectiveness to LP. The primary outcome is the Japanese Orthopedic Association score. The non-inferiority margin for the primary outcome is five. The Ethics Committee of Fujian Medical University Union Hospital, Fuzhou, China, has approved this study (project number: FJMUUH05). Any results of the trial will be published in international peer-reviewed journals and disseminated through presentations at scientific conferences.

**Trial registration number:**

ChiCTR2100047582

## 1. Introduction

Although spinal intradural extramedullary (IDEM) tumors grow slowly, the spinal canal is limited by its small volume, and the spinal cord and nerve roots are compressed or invaded by the tumor, which causes sensory, motor, and autonomic dysfunction (1). The treatment of spinal IDEM tumors focuses on removing tumor tissue and decompressing the nerve root and spinal cord to improve nerve function (2, 3). Traditional surgical methods include laminectomy (total laminectomy and hemilaminectomy), laminotomy, and laminoplasty (LP) (4). Among the available spinal surgeries with different indications, a “standard surgical procedure” for treating spinal IDEM tumors has not been determined. Owing to the development of minimally invasive spine surgery (MISS), the paraspinal mini-tubular technique (PMTT) has been introduced for treating IDEM tumors (5–7). Most studies reported that tubular spine surgeries reduced the volume of estimated blood loss (EBL) and shortened the length of hospital stay. The postoperative outcome of the patients was good, with fewer complications. These together promoted the development of MISS. A microscope-assisted PMTT has a larger operating space than endoscopy and a relatively wide range of indications, including various types of intervertebral disc herniation, spinal stenosis, spinal tumors, and spinal cord injuries (8). With the popularization and application of PMTT, the safety and effectiveness of treating spinal-related diseases have been increasingly recognized. Compared with traditional surgery, the microscope-assisted microtubular technique has the advantages of less tissue damage, fewer complications, shorter length of hospital stay, reduced postoperative pain, improved surgical efficiency, and reduced risk of spinal instability (9–11). To the best of our knowledge, prospective, randomized controlled trial studies with a large sample comparing the safety and effectiveness of the PMTT and LP in the treatment of spinal IDEM tumors, have not been conducted. Therefore, this study aims to explore the difference in the therapeutic effect of the PMTT and LP in the treatment of patients with spinal IDEM tumors through a prospective, multi-centered, non-inferiority randomized clinical trial. The main hypothesis is that PMTT has advantages in cost-effectiveness, and it is equivalent in safety and efficacy to LP.

## 2. Methods and analysis

### 2.1. Study description

**Figure 1:**
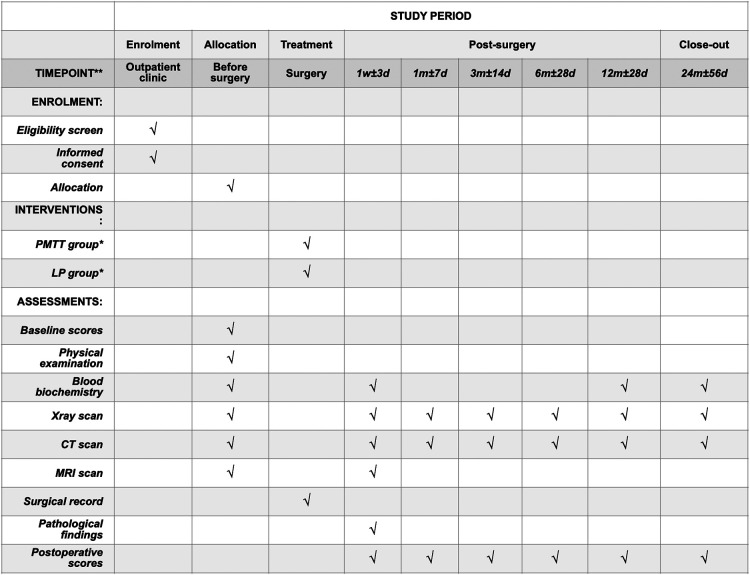
The schedule of enrolment, interventions, and assessments. *PMTT group, paraspinal mini-tubular technique; LP group, laminoplasty group.

The FJMUUH05 trial is designed as a prospective, multi-center, non-inferiority RCT with two parallel groups in eight hospitals from seven provinces in China. A total of 280 patients aged 10–75 years with confirmed radiographic diagnosis and IDEM tumor lengths of less than three segments will be enrolled in this study. After the patients sign a written informed consent to participate, they will be randomized to one of the two groups: the PMTT group, which will receive paravertebral approach and microtubular technique treatment, and the LP group, which will receive laminoplasty treatment. The follow-up period will last for two years. The study is registered at https://www.chictr.org.cn, which can be accessed online (ChiCTR2100047582). Consent (version date January 21, 2021, V.1.0) will be obtained by an investigator who will comply with applicable regulatory requirements and adhere to the ethical principles of the Declaration of Helsinki. The time points, randomization, treatment allocation, and assessment are summarized in Figure 1.

### 2.2. Participant eligibility and recruitment

Participating surgeons and hospitals must satisfy the following criteria to prevent bias: surgeons must have experience performing >100 surgeries for spinal tumors, participating hospitals should have performed >40 spinal tumor surgeries annually, and they must have completed the Fujian Medical University Union Hospital spinal training program.

Eligible patients with spinal IDEM tumors will be referred by the participating surgeons, following a formal outpatient assessment. This study follows the informed consent principle; thus, each patient must provide written informed consent prior to group randomization. Detailed inclusion and exclusion criteria are listed in Table 1.

**Table 1:** Selection criteria for trial eligibility.

Inclusion criteria:
Age 10–75 years
Spinal IDEM tumors confirmed by clinical symptoms and radiographic findings
Magnetic resonance imaging with the length of the tumor less than three segments
The patient has given oral and written informed consent to participate
Exclusion criteria
Spondylitis or degenerative spondylolisthesis
Previous surgery on the same or adjacent spinal level
Severe somatic or psychiatric illness
The patient fails to be present in the follow-up appointments required by the protocol, or the investigator deemed that the risk of patients in the study increases
The patient cannot provide written informed consent or cannot follow the trial
The survival period of the patient is <1 year

### 2.3. Patient and public involvement statement

The patients and public are not involved in the design, conduction, reporting, or dissemination plans of this research.

### 2.4. Randomization and blinding

Once written informed consent is obtained, patients would be randomized to undergo either the paravertebral approach or microtubular technique, or LP in a ratio of 1:1. The patients will be randomized using a block randomization model (block size: six). Computer-generated random number tables will be prepared by an experienced statistician. After baseline data are obtained, and a physical examination has been conducted, allocation of treatment will be performed by the computer system, and the results of the allocation will be provided to the surgeon in a concealed envelope one day before surgery. Due to the noticeable differences between the PMTT and LP procedures, blinding of the patients will not be strictly required. However, all researchers and data analysts will be blinded to the allocated intervention during the follow-up period of 2 years.

### 2.5. Treatment

Patients will be randomized to receive either PMTT or LP treatments. The tumor's location will be verified using a mobile image intensifier with fluoroscopy (anteroposterior and lateral views). The PMTT and LP will be performed under general anesthesia. All the patients will be placed in a standard prone position. The surgeons involved in this study would have had extensive experience with both procedures.

#### 2.5.1. (A) intervention: PMTT

After inducing general anesthesia, the patients will be placed in the prone position to minimize lumbar lordosis or thoracic kyphosis and to avoid compressing the abdomen. A 20–25 mm skin incision will be made approximately 20–35 mm lateral to the midline (adjusted according to the patient's body habitus), and the accurate target level will be confirmed by lateral fluoroscopy. The paravertebral approach will involve the paraspinous muscles, and the muscles will be bluntly dissected by a muscle-splitting technique. After the smallest dilator is inserted to reach the lamina or peripheral bone structure, the dilators will be piled sequentially, and the working tubule (diameter, 14 or 16 mm) will be inserted over the dilators. The dilators will be removed, and a tubular surgical channel will be established. The tubule will be fixed using a flexible fixed arm mounted on the operating table. The tubule could be angulated to expand the operating field because of the flexible fixed arm. The subsequent procedure will be performed under a microscope (OPMI Pentero, Carl Zeiss, Germany). Part of the lamina will be removed using high-speed drills and Kerrison rongeurs. After the dura mater is incised, the IDEM tumor will be separated and removed piece by piece using a microsurgical technique. The dura mater will be sutured tightly. After the tubule is withdrawn, the paravertebral muscles will be repositioned, and the fascia, subcutaneous tissues, and skin will be sutured in sequence. Thereafter, a surgical drain will be placed in the resection site for 24–48 h.

#### 2.5.2. (B) intervention: LP

A skin incision will be made, and subcutaneous tissue, lower back fascia, and muscle will be incised in layers to expose the spinous process and lamina of the spinal segment where the tumor is located. The retractor will be used to expose the bone structures, and the complex of the spinous process and lamina will be removed using an ultrasonic bone knife combined with a high-speed drill. The ligamentum flavum will be removed, and the dura will be sharply incised. The tumor will be separated and removed with the aid of a microscope, and the dura will be closed with a 5.0 or 6.0 suture. The spinous process and lamina will be repositioned, and the laminoplasty will be performed with titanium screws and connecting plates. The paravertebral muscles will be repositioned, and the fascia, subcutaneous tissue, and skin will be sutured one after the other. A drain will be placed at the resection site for 24–48 h.

### 2.6. Baseline assessment

The demographics, medical history, family history of tumors, results of a physical examination, body mass index, tumor location, visual analog scale (VAS) scores, and Japanese Orthopedic Association (JOA) scores will be recorded at baseline. Data will be collected prior to randomization.

### 2.7. Withdrawal

All patients can decide to withdraw at any time. If a patient withdraws, information will not be recorded in the study. However, the research team can still collect the outcome data from the healthcare records.

### 2.8. Outcome measurements

Patients must have an indication for the surgery. The primary outcome variable is the JOA score. The secondary outcome variables include the VAS score, physical examination findings, length of hospital stay, expenses, and complications. Outcomes will be measured on the first postoperative day, and thereafter at the 1st week, and at 1, 3, 6, 12, and 24 months after surgical treatment. A physical examination will be conducted after 1 week, 1 month, and 12 months from the day of the surgery (Table 1). The non-inferiority margin for the primary outcome is five.

### 2.9. Primary outcome

We will use the JOA score to assess the primary outcome (12, 13). It is divided into the cervical and JOA scores, including subjective symptoms, clinical signs, daily activities, and bladder function. Each item had one or more problems. The total score of the JOA was 29, and the total JOA score of the cervical spine was 17. The lower the score, the more obvious the dysfunction. The outcome parameters will be assessed using validated questionnaires and physical examination. Data from the questionnaires will be collected at 1 week and at 1, 3, 6, 12, and 24 months after the surgery by the research nurse.

### 2.10. Secondary outcome

Secondary outcomes include the gross tumor resection(GTR) rate, tumor recurrence rate, VAS, assessment of back and leg pain at rest, physical examination, length of hospital stay, expenses, and complications. One of the secondary outcomes was the VAS of pain. Perceived pain intensity was measured using the VAS score [pain scale ranging from 0 (no pain) to 100 mm (the worst pain imaginable)] (14). The pain will be assessed because most patients have pain in different regions. A physical examination will be conducted at 1, 6, and 12 months after the surgery. This will include the sphincter function test, muscle strength exercise, patellar and Achilles tendon reflex assessment, and sensory assessment of the affected spinal region. Muscle strength will be scored on a scale ranging from zero (no contraction) to five (normal muscle strength) (15).

The cost-effectiveness of the hospital stay will be evaluated by summarizing the cost of the surgical procedure and the number of days before discharge. The cost of the additional procedures in case of complications will be added to the total cost of the patient's hospital stay. The primary cost of treatment will include the cost of hospital admission, surgery, medication, rehabilitation, and other healthcare utilization. The details of these charges will be registered in a diary. Immediately after the operation, a systematic assessment of complications (including cerebrospinal fluid leakage, venous thromboembolism, wound infection, urinary tract infection, hematoma, and progressive neurological deficit) will be conducted by the surgeon and research nurse until patient discharge. Surgical data will include intraoperative spinal cord and nerve root injury, operative time, and intraoperative blood loss.

#### 2.10.1. Adverse and serious adverse events

All adverse events related or not to the interventions will be reported during the complete study period. The list of adverse effects is adjacent segment instability, surgical site infection, worsening neurological symptoms, pain recurrence, dural tears, and CSF leakage.

### 2.11. Sample size calculation

The sample size for this study was calculated based on the JOA scores. Based on the preliminary data of our department, the mean difference and SD for the JOA used in the sample size calculation were as follows: mean 21.7, SD 5.8. The sample size for non-inferiority trials was calculated using the following: (1) significance level (alpha) of 0.05 and (2) power (beta) of 90%. We estimated that 112 patients would need to be included in each group. Accounting for 10% attrition and the actual situation of each participating center, 280 patients will be recruited in total. We intend to recruit patients within two years. Recruitment for the study began in October 2021.

### 2.12. Statistical analysis

All data will be analyzed according to the intention-to-treat principle (16, 17). The baseline data will be compared and analyzed using descriptive statistics [mean (SD), proportion, or median (range)] to determine whether balanced groups were obtained after randomization. Student's *t*-test or Mann–Whitney's *U* test will be used to compare continuous variables. Categorical variables will be compared using *χ*^2^ or Fisher's exact tests. All comparative analyses will be reported with point estimates [means (SD) or odd ratios (ORs)], 95% CIs, and *p*-values. Statistical significance will be set at *p *< 0.05. Non-inferiority margins will be set as listed in Table 2. Statistical analysis will be performed using appropriate statistical software (e.g., SPSS version 22.0 or Stata).

**Table 2:** Non-inferiority margins.

Outcome measurements	Expected differences	Non-inferiority margin
JOA	<5	5
VAS	<5	5
muscle strength test	<5	5
Sphincter function test	<5	5

### 2.13. Ethics and dissemination

The study will be performed in accordance with the Declaration of Helsinki. This article describes a protocol for a prospective, non-inferiority randomized controlled trial to examine the safety, efficacy, and cost-effectiveness of PMTT vs. LP in treating spinal IDEM tumors. This study was approved by the Ethical Committee of Fujian Medical University Union Hospital, Fuzhou, China, in June 2021(2021YF022-01). Informed consent will be obtained from all eligible participants prior to randomization. The results of the research will be published in international peer-reviewed journals and disseminated through presentations at scientific conferences.

## 3. Discussion

The FJMUUH05 trial is a multicenter RCT, the first RCT of surgery for spinal IDEM tumors. To date, similar RCTs have not been reported on this topic. The trial was approved by the Chinese Clinical Trial Registry (ChiCTR, www.chictr.org.cn). The trial hypothesis is that PMTT has superior cost-effectiveness and is equivalent in safety and efficacy to LP for treating spinal IDEM tumors.

Tubular spinal surgery has gained significant prominence because of the development of minimally invasive technologies and instruments. The tubular retractor is inserted to reach the operation field by separating the paravertebral muscle and exposing the pathological lesions. This technology allows surgeons to remove tumors with two-handed operations and simultaneously use multiple microscopic instruments. Moreover, intraoperative three-dimensional imaging is another massive shift from endoscopic technology, which lets the operator conveniently operate the imaging apparatus, thereby reducing the complication rates of dural rupture. Compared with traditional surgery, fewer clinical studies have been conducted on the selection of surgical indications. The safety and effectiveness of the operation and the treatment of complications during and after the operation lack a unified opinion. The patients will be randomized to the PMTT or LP groups after tumor resection. The trial aims to study the clinical results of PMTT vs. LP for spinal IDEM tumors. This RCT study provides high-quality evidence for the efficacy of tubular spine surgery for spinal IDEM tumors. We believe that the FJMUUH05 trial will provide useful information on the long-term effects of the two treatments for spinal IDEM tumors. To date (October 2021), 11 patients have been included in the trial.

In conclusion, the FJMUUH05 trial is a multicenter RCT investigating the cost-effectiveness, safety, and effectiveness of PMTT and LP in spinal IDEM tumors. When this trial is completed with the hypothesis confirmed, it will popularize the application of PMTT and improve patient outcomes.
